# Impact of COVID-19 pandemic on health care workers (HCWs) in Sindh Province of Pakistan

**DOI:** 10.1186/s12961-023-01022-5

**Published:** 2023-07-31

**Authors:** Saifullah, Zhiqiang Ma, Mingxing Li, Muhammad Qasim Maqbool

**Affiliations:** 1 School of Management, Jiangsu University, Zhenjiang, 212013 China; 2 Department of Management Sciences, University of Okara, Renala, Okara, 044 Punjab Pakistan

**Keywords:** HCWs, Physical and psychological health, Performance, Fear of infection and stress

## Abstract

**Background:**

In Pakistan, the COVID-19 outbreak posed a significant challenge for healthcare workers in the country’s public hospitals. The HCWs faced several problems in terms of the COVID-19 pandemic. Therefore this study investigated how the COVID-19 pandemic has affected the medical staff at the public hospital in Sindh Province, Pakistan.

**Methods:**

In this study, a qualitative exploratory design was used. Semi-Structure interviews (SSI) were conducted by using an open-indeed questionnaire (OIQ) for data collection. An inductive approach was used for theoretical data analysis. A total of 320 HCWs participated to complete the criteria of the study from 10 different public hospitals.

**Results:**

The study result showed the Sindh public hospital’s insufficient infrastructure, lack of health protective equipment, shortages of isolation rooms and beds, and emergencies during the COVID-19 pandemic caused HCWs to experience physical and psychological weariness, sleep disturbance, mental stress, and fear of infection.

**Conclusion:**

The study concluded that public hospitals’ insufficient infrastructure, furniture, emergency wards, and safety equipment during the COVID-19 pandemic significantly damaged HCWs’ physical and psychological health, generating fear of infection and sleep disturbance. Additionally, Sindh healthcare workers’ fear of illness and isolation may impair family connections.

## Introduction

In December 2019, China released its first COVID-19 pandemic report [1]. WHO entitled it as a (“COVID-19”) on 11th February 2020 [2]. After January, this virus was labeled a global pandemic and a public health emergency [3]. Approximately, 7 553 182 people were infected with COVID-19 globally on (June 13, 2020), and 423 349 people deaths resulted due to the COVID-19 battle [4]. Moreover, the COVID-19 pandemic caused high psychological fear among healthcare workers [5, 6]. Pakistan is a middle-income nation with a subpar healthcare system and Pakistan is susceptible to COVID-19 [7]. The healthcare crisis has resulted in a spreading pandemic in developing nations such as Pakistan [8], such as 15th July 2020, a total of 255 769 cases were reported with 5386 deaths [9]. As high stress in this period, the COVID-19 outbreak in Pakistan served as a harsh wake-up call to the country’s deficient health system [10].

On February 26, 2020, Pakistan announced the first case of COVID-19 in Karachi, Sindh Province, By June 14, 2020, 139 230 cases had been confirmed, and 2632 fatalities had been reported [11]. The second wave of COVID-19 spell on October 28, 2020, in Pakistan when the daily increase in cases reached 750, up from 400 to 500 just a few weeks ago. Across the country, there was a sudden increase in active cases from 6000 to 11 000, as well as hospital admissions with 93 critical cases on ventilators [5]. A third-wave new SARS-CoV-2 variant from the United Kingdom (also known as 20I/501Y.V1, VOC 202012/01, or B.1.1.7) was discovered [7] and has been found in over 64 nations, including Pakistan, as of January 27, 2021 [8]. With an average of 100 patients dying each day in Pakistan, this B.1.1.7 variant is linked to a higher risk of death than other variants [9].

**Fig. 1:**
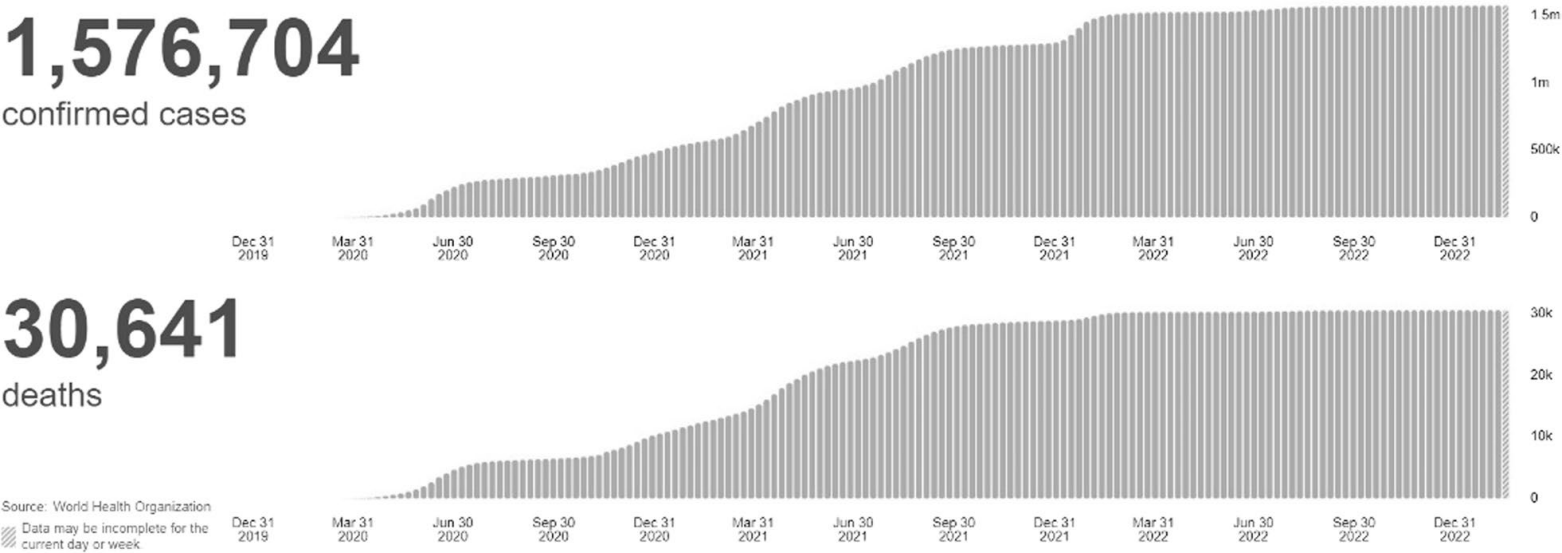
Pakistan situation of COVID-19 related cases and deaths

Figure 1 shows there are 30 641 deaths and 1 576 704 confirmed cases of COVID-19 in Pakistan from 3 January 2020 to 4:24 PM CET on February 21, 2023 [12]. The country has gone through four waves, each with a different variant, most recently Delta [13].

Pakistan’s healthcare delivery system is complicated because it competes with formal and unofficial private-sector healthcare systems as well as healthcare subsystems run by the federal and provincial governments [14, 15]. The country’s healthcare system is also characterized by disparities in healthcare delivery between urban and rural areas and a shortage of health managers, nurses, paramedics, and skilled birth attendants in the outlying areas [16, 17]. In 2015, Pakistan had 1167 public hospitals, with a hospital bed-to-population ratio of one for every 1613. (WHO recommendation of bed-population ratio: 5 per 1000 population) [18]. The World Health Organization (WHO) reports that Pakistan ranks 122nd out of 191 nations for its healthcare quality system and Health Care Professions [19, 20]. In addition, Healthcare professionals are on the frontline for COVID-19 patients’ treatment in which they can be infected [21].

The documentary record shows that a large number of quantitative studies on COVID-19 infection and death ratio were conducted in Sindh Pakistan. A qualitative study is missing in the study regions, especially on healthcare workers who are caring for patients during term of the respiratory pandemic. Table 1 represents provincially disaggregated data for the four provinces and other territories. It shows the highest number of cases has resulted in the Sindh Province. In addition, the Government of Sindh (2020) Daily Situation Report, August 2020 reported that approximately 1804 HCPs, including 1626 doctors and 178 nurses, were infected with the COVID-19 virus in Sindh [11]. Given these reasons, the goal of this study was to find out how the COVID-19 pandemic has affected HCWs in public hospitals in Pakistan’s Sindh Province.

**Table 1:** Provincially disaggregated data as of July 28, 2020, table-3 [22]

Region	Confirm cases	Active cases	Deaths
AJK	2055	462	50
Baluchistan	11 654	1438	136
Gilgit-Baltistan	2042	334	50
Islamabad	14 963	2421	165
PKP	33 724	4814	1186
Punjab	92 452	7807	2133
Sindh	119 398	8237	2172
Overall Pakistan	276 288	25 513	5892

## Background

### HCWs infection and deaths with COVID-19 worldwide

Data on healthcare worker infection and death are not reported by every country. A recent study found healthcare workers infected and dying as a result of the pandemic in several countries.

**Fig. 2:**
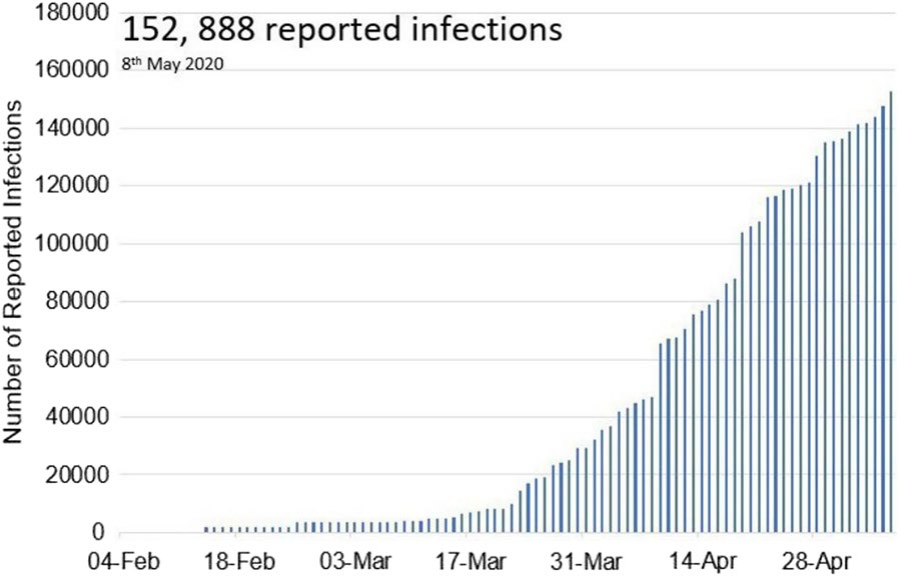
Globally HCWs infection due to COVID-19

**Fig. 3:**
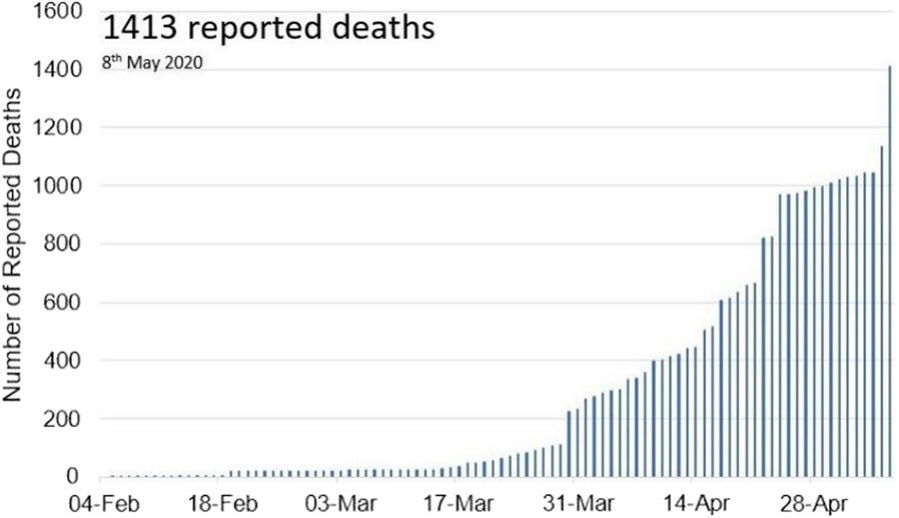
Globally HCWs death due to COVID-19

Figure 2 shows that a total of 152 888 HCWs had been reported to have COVID-19 infection as of May 8th, 2020. This represented 3.9% of the 3 912 156 COVID-19 patients in the world as a whole. A total of 130 countries reported at least one case of COVID-19 infection in healthcare workers ([23], figure-3).

**Fig. 4:**
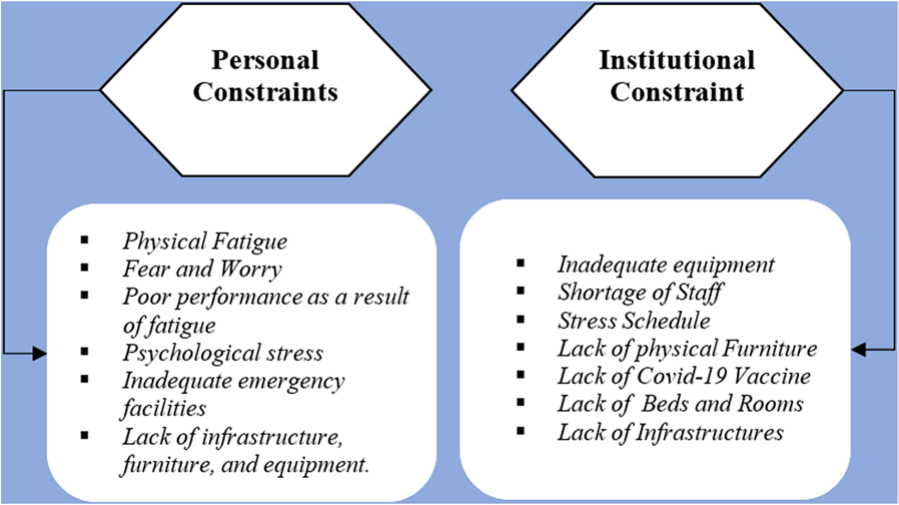
Hospital condition and health care workers (HCWs) personal constraints

Figure 3 shows that as of May 8, 2020, there had been 1413 reported HCW deaths. This means that for every 100 HCWs infected, one died. This accounted for 0.5% of the total number of COVID-19 deaths worldwide (270 426). It should be noted that 2922 COVID-19 deaths were reported in countries where no data on COVID-19 deaths among healthcare workers were available. As of 8 May 2020, 67 countries had reported at least one COVID-19-related HCW death (Fig. 4) [23].

### Psychological effects of COVID-19

Numerous documentaries report that subjects with psychological symptoms are likely emotional disturbance [24]. In a study conducted by Zhu et al. on a total of 5062 HCWs to assess the psychological impact of COVID-19, the authors found 29.8% of stress, 24.1% anxiety, and 13.5% depression [25]. Similarly, authors found that HCWs who had direct contact with COVID-19 patients were more likely to experience anxiety [26]. Also, Lu et al. studies of frontline workers found that medical staff had higher levels of fear, anxiety, and depression than administrative staff [27]. A recent study called “Healthcare Workers’ Mental Health in Pandemic Times: The Predictive Role of Psychosocial Risks” found that supporting network development at work is important to keep healthcare workers from feeling emotionally stressed and to improve their mental health [28].

### Anxiety and loneliness impact of COVID-19

According to the findings of a study, feelings of uncertainty and fear of infection are associated with social isolation, which is linked to restrictions and lockdown measures, this worry causes higher levels of anxiety [29]. Also, the authors report that anxiety is linked with fatigue and decreased healthcare worker performance, and boredom, loneliness, frustration, anger, and suffering are caused by quarantine restrictions [30]. Besides, in terms of pandemic time more tragic effects associated with pervasive anxiety may include poor social support, loss of freedom, separation from dearests**,** boredom, and uncertainty [31]. Women utilize the internet more than men to research COVID-19-related infection management, according to a recent Times study titled “Comparison of Safety and Health Risk Perceptions toward COVID-19 Pandemic Based on Gender in Korean University Students’ Work While Studying” [32]. According to the article “Influencing Factors of High Post-Traumatic Stress Disorder (PTSD) among Medical Staff during COVID-19: Evidence from Both Meta- and Subgroup Analysis,” (PTSD) is greater among healthcare personnel (PTSD) [33].

### Physical impact of COVID-19

Critical events and pandemics may have varied effects on people’s physical activity and mental health at various levels of society, including patients, healthcare professionals, and families [34–36]. Similarly, the emergency, along with both COVID-19 stress and social isolation, may have emotional and physical consequences [37, 38]. Besides, it has been well-established that regular physical activity positively affects health and well-being [39].

### Suicidal and ideation impact of COVID-19

Many studies show that suicide death is associated with mental health disorders. According to the author’s reports that suicide attempts and completed suicide rates have increased during the hard times of COVID-19 pandemic [40, 41]. Additionally, there is a high rate of suicide attempts and suicidal ideation among medical professionals [42]. It is important to note that when the COVID-19 pandemic was at its worst, the risk of suicide among healthcare workers increased due to increased levels of psychological distress, the deaths of COVID-19 patients, a lack of feelings of control, self-blame for patients’ helplessness, and increased working hours [43]. Additionally, healthcare personnel has committed suicide in other nations, including the United States, England, Italy, Mexico, and India [44–46].

### Loneliness impact of COVID-19

According to research, social isolation, and loneliness, both have negative effects on people’s quality of life [47, 48]. Therefore, it has been urged study to look into how loneliness affects the general populace during the COVID-19 lockdown [49]. The research has concentrated on the psychological effects of COVID-19 on healthcare workers such as nurses, clinical technicians, and doctors [25, 50]. According to a recent study, 27% of hospital nurses’ sick leave was caused by high work speed, sleep issues, getting a viral or bacterial infection from patients or colleagues, poor staffing, and high physical exertion [51].

### Family relations impact of COVID-19

Global human personal life and family life have been badly impacted by COVID-19 pandemic. In most cases, HCWs are worried about contracting COVID-19 and spreading it to family members at home [52–59]. HCWs who lived with children or older relatives were especially worried [60–64]. After reviewing the literature, it was discovered that the majority of HCWs faced several types of issues due to the pandemic. However, In Pakistan COVID-19 related studies were conducted about the number of infections and death ratio of the patients. There is a missing gap in the study about healthcare workers. Therefore, this study attempted to investigate the impact of COVID-19 on healthcare workers in Pakistan’s Sindh province. This is because the first COVID-19 case and a large number of cases have resulted in Sindh province. Additionally, Sindh province is Pakistan’s second-most populous province and its commercial center.

## Methods

The qualitative exploratory method was applied in this study. Based on the study nature, a semi-structured interview (SSI) was used to explore healthcare workers’ experience during a hard time of the pandemic. The authors express that qualitative investigation provides more detailed and comprehensive information about issues that may have been missed by a survey-based research method [65]. According to the author, interviews are used once only with individuals or with a group to cover 30-min interviews [66]. The semi-structured interview is based on the interview guide which appearances of questions to explore the interviewer [67]. Moreover, interview recording is an easier way to focus on verbal content to promote and enables transcription to get a verbatim record of the interview [68]. Hence, we used this research method to find out core responses from HCWs.

### Study design and setting

The participant included HCWs who were working in the public hospital sector in the Sindh province of Pakistan. The COVID-19 first case resulted in this province and a large number of infections and death have resulted in this region, therefore, were chose this region. Besides, In Pakistan, public hospitals under comes the provincial authority. Hence the data were collected only from the Sindh Province of Pakistan. A total of 10 public hospital HCWs participated (Table 3). Participants allowed us to take interviews face to face during the break time by using masks and distancing. The interview was conducted in the hospital’s meeting room this is because the meeting hall was already well-arranged in the hospitals, so the hospital’s meeting hall was used to save the participants’ time. The researcher thanked and admire all participants for their time and participation in the interview. They were guaranteed that responses will be used top-secret and data will be only used for the research work.

### Data collection

We used semi-structured interviews to achieve rich experiences of HCWs to full fill aim of the study. Therefore, open-ended questions were utilized in the current study. We used a non-probability sampling technique called purposive sampling. Because the study sought the core responses of healthcare workers. The study include major themes (1-Physical Health during COVID-19; 2-Stress during COVID-19; 3-Performance during COVID-19; 4-Psychological Health during COVID-19; 5-Emergency Facility during COVID-19; 6-Sleeping Mood during COVID-19; 7-Infrastructure and Physical Furniture during COVID-19). The interviews were conducted by three Ph.D. scholars two are the authors of this study. We carry out a maximum number of interviews group-wise face-to-face. Each group contained 10 people. We began by introducing ourselves and the reasons for the interview, and then we moved on to the introduced sections. Please tell us about your personal information. After personal information, we started probing question sections such as Please tell us about your physical health during the pandemic’s difficult period. What caused you to become physically exhausted during the pandemic? The questions were asked in a manner during the interview. The interview summary is given in Table 2 and the respondents’ personal information is in Table 3. All interviews lasted at least 30–50 min and were done in Urdu, Pakistan’s native language. After a successful interview, we analyzed it textually and summarized it for final validation. The participants were engaged and admired for their desire to share their pandemic experiences. All interviews were performed from 5th August to 25th September 2021.

**Table 2:** Interview summary

Interview summary
1. How were you feeling physically at work during the difficult COVID-19 pandemic?
2. How did you feel mentally at work during the challenging COVID-19 pandemic?
3. How did you do professionally at work amid the challenging COVID-19 pandemic?
4. How did you feel about resting after being off duty during the difficult COVID-19 pandemic?
5. How did your family get along during the difficult COVIDian-19 pandemic?
6. How did the physical infrastructure furniture of hospitals do during the difficult COVID-19 pandemic?
7. How was the fear of infection and stress while working in the hospital during the pick time of the COVID-19 pandemic?
8. How effective was your experience during the hospital’s emergency quarantine unit during the difficult COVID-19 pandemic?

**Table 3:** Participator’s personal information (*N* = 320)

Personal information	Frequency	Percentage %	Mean	Std. deviation
Gender			1.4375	0.49686
Male	180	56.3		
Female	140	43.8		
Age			2.3688	0.92083
Between 20 and 25 years	60	18.8		
26–30 years	114	35.6		
31–35 years	120	37.5		
36–40 years	20	6.3		
Above 40 years	6	1.9		
Experience			2.8281	1.15499
1–5 years	50	15.6		
6–10 years	75	23.4		
11–15 years	95	29.7		
16–20 years	80	25.0		
Above 20 years	20	6.3		
Hospitals names			4.5500	2.80125
Hospital-A	50	15.6		
Hospital-B	55	17.2		
Hospital-C	32	10.0		
Hospital-D	40	12.5		
Hospital-E	25	7.8		
Hospital-F	30	9.4		
Hospital-G	20	6.3		
Hospital-H	23	7.1		
Hospital-I	25	7.8		
Hospital-J	20	6.3		
City names			2.7250	1.68322
Karachi	110	34.4		
Hyderabad	60	18.8		
Sukkur	50	15.6		
Nawabshah	34	10.6		
Larkana	40	12.5		
Kamber	26	8.1		
Total	320	100.0		

Table 2 shows the interview summary and the types of questions that were asked to HCWs during the interview time.

### Analysis

We used the interview analysis method of thematic analysis [69]. All interviews were transliterated from Urdu to the English language. Each interview’s transcriptions were made within 24 h. All transcription was revised line by line twice for the study’s validity. Each transcription was textually analyzed during revision. The first transcription listened whole then each question was listed twice, and then each theme question was analyzed using notebook coding. The coding was done with own understanding: supportive (COVID-19 affected); not supportive (not affected); highly supportive (highly affected); and extremely not supportive (nothing affected during the pandemic). Additionally, each group’s interview was analyzed on the same day. Analyzing after the interview was easy. For study validity and bias reduction, analyses were done on the same day. This approach was used for interview analysis for internal validation. Additionally, agreements were shared and signed by all the study members. The survey interviewed 330 HCWs. 10 of the 330 participants were excluded due to incomplete interviews due to time constraints. Out of 330, a total of 320 HCWs interviews were completed at 10 different public hospitals in Pakistan’s Sindh province. The participants and hospital details are given in Table 3.

## Results

Table 3 shows the total of (*N* = 320) HCW interviews conducted from 10 public hospitals in the Sindh Province of Pakistan. The (*N* = 180) Men and (*N* = 140) Women participated in the interview. The age of the respondents was (*N* = 60) between 20 and 25 years, (*N* = 114) between 26 and 30 years, (*N* = 120) between 31 and 35 years, and (*N* = 20) respondents’ age between 36 and 40 years, and only (*N* = 6) respondents above 40 years older. In light of these workers’ experience, the (*N* = 50) of 1–5 years experienced, (*N* = 75) persons with 6–10 years of experience, (*N* = 95) persons with 11–15 years experienced, (*N* = 80) persons with 16–20 year’s experience, and only (*N* = 20) persons had more than 20 years of experience. Moreover, all these participants were from different public hospitals and cities. The name of the hospitals was strictly prohibited to mention due to ethical commencements. Therefore, we used hospitals’ names A–B–C wise. The Hospital-A (*N* = 50) respondents, Hospital-B (*N* = 55) respondents, Hospital-C (*N* = 32) respondents, Hospital-D (N = 40) respondents, Hospital-E (*N* = 25) respondents, Hospital-F (*N* = 30) respondents, Hospital-G (*n* = 20) respondents, Hospital-H (*n* = 23) respondents, Hospital-I (*n* = 25) respondents, Hospital-J (*N* = 20) respondents.

Moreover, these data were collected from major cities of Sindh province of Pakistan. A total of (*N* = 110) respondents from Karachi City, (*N* = 50) respondents from Sukkur city, (*N* = 60) responded from Hyderabad City, (*N* = 34) respondents from Nawabshah and (*N* = 40) respondents from Lankan City, and (*N* = 26) of respondents from Kamber City. The study themes and HCW’s interview results are given in Table 4.

**Table 4:** Themes and interview results

Theme-1: physical health during COVID-19
The HCWs expressed that during the pandemic, prolonged work with protective gear like gloves, masks, and protective clothing caused physical discomfort and exhaustion
Theme-2: stress during COVID-19
Participants expressed that working in hospitals during the COVID-19 pandemic resulted in stress and fear of infection. Additionally, they noted that during the epidemic, conducting duties to treat patients was extremely stressful for oneself and colleagues
Theme-3: performance during COVID-19
The HCWs stated that in terms of COVID-19, the increased fear of infection has had a negative influence on work performance. They stated that while working on the patient’s treatment, they were terrified of infection and were unwilling to work in such a dangerous condition. Furthermore, they stated that the usage of fully protective clothing created fatigue and difficulty in performing the job
Theme-4: psychological health during COVID-19
Participants said that working in the hospital during the pandemic’s tumultuous period induced extreme dread of infection, and feelings of isolation, which were harmful to mental health. Moreover, throughout the patient’s treatment, a sense of self-infection anxiety, its influence on a family member, and the patient’s isolation had a greater unfavorable effect on psychological health
Theme-5: emergency facility during COVID-19
Most of the participants stated that the emergency facility was inadequate as a shortage of rooms, beds, and related COVID-19 medicine. Further, they expressed that while the emergency rooms and beds are in shortage for present patients’ treatment, in this case, HCWs getting infections could be terrible to handle the COVID-19 pandemic treatment process in the public hospital. The study found unavailability of Emergency rooms and beds facilities in the public hospital of Sindh during a hard time of the COVID-19 pandemic
Theme-6: sleeping mood during COVID-19
Healthcare workers report having trouble sleeping during the peak of the COVID-19 outbreak. During sleep, you may have thought about lonely patients, blamed yourself for failing to ensure the patients’ safety, worried about catching an illness, or reflected on the love you feel for another person. HWCs also reported that the negative effects of loneliness, such as not getting together with loved ones, had an impact on their ability to fall asleep
Theme-7: infrastructure and physical furniture during COVID-19
The HCWs expressed that during the pic time of the pandemic, the public hospital buildings and rooms were short. In term of lack of rooms and beds caused difficulty to treat the patient. Further, they expressed a lack of furniture and other equipment for COVID-19 made a big problem in handling treatment with the patient and self-care in the public hospital sector in Sindh province of Pakistan

## Discussion

Safety and security in the health field, taking into account the basic rights of HCWs and Patients in the public hospital sector. In Sindh province’s public hospital, the work of HCWs is thought to be very dangerous because COVID-19 shows that they are more likely to get sick because of their lack of health care personal protective equipment. In terms of pandemic emergency medical staff, they were not well trained; additionally, pandemic emergencies were new, and medical staff was concerned about patent treatment and self-health due to the entire difficult experience of respiratory diseases.

As a result of Pakistan’s lack of medical facilities during the early stages of COVID-19, questionable samples were transported to China [70]. In this critical situation, healthcare workers getting fear or sick was a big issue. The healthcare workers’ staff shortage and getting sick healthcare workers could be more worried for medical staff and the state to control the pandemic and play the treatment process. Such as stressful events make healthcare workers more likely to get sick [71, 72], and fear and uncertainty caused by the pandemic can make family or interpersonal violence or conflict worse [73, 74]. The healthcare workers’ staff faced various types of fear and stress, such as infection fear during patient treatment or infection from staff; in addition to all of this, healthcare workers returning home were a frightening experience for their family life.

Also, healthcare workers who wear protective clothes probably follow the standard operating procedure (SOP) of wearing a mask and heavy protective clothes, headgear, and gloves, and doing their jobs with fear of getting an infection, since most public hospital workers were very busy. Because due to fear of infection and following standard operating procedures (SOP), HCWs used the protective equipment for longer hours caused them so tired and fatigued.

Healthcare workers say that fear of infection, self-blame for not helping the patient, poor sleeping mood, and negative family life have a major impact on psychological health. According to the authors, People who perceive the risk of infection because of the developing of risk infection, worried, and fear for his/her health and others including fear of infection among family members [75–77]. Similarly, healthcare workers’ high workload negatively impacts their family life such as burnout and distress in family relationships, and marital complications [78, 79].

Similarly, healthcare workers’ performance is negatively impacted by physical fatigue, psychological fatigue, and fear of infection. There is no doubt that fear of infection and huge physical-mentally fatigue can be caused fatigue performance. According Deloitte survey of Chinese enterprises revealed that 46% of them expect a decrease in performance as a result of COVID-19. Pakistan is considered an underdeveloped country with a lack of healthcare resources whereas COVID-19 was one of the biggest challenges for state and healthcare professionals. Besides in terms of emergencies of respiratory no one was fully prepared to control them. Similarly, there is no doubt in it that healthcare workers faced a lack of emergency facilities like poor infrastructure, furniture, and personal protective equipment during the pic time of the pandemic.

The HCW’s work considering highly risky in terms of COVID-19. The work of public hospital healthcare providers is viewed as extremely hazardous due to the widespread concern that COVID-19 could spread through the facility’s poor safety equipment and emergency facility. In addition, working long hours while worrying about contracting an illness is a stressful and tiring combination. A lack of healthcare workers and infrastructure, such as buildings, beds, rooms, and safety equipment, makes it so that HCPs can rest in the face of emergencies and the fear of infection that comes with treating a large number of COVID-19 patients in public hospitals.

## Conclusion

Protection for healthcare workers and patients in the community hospital setting, with due consideration for their fundamental rights. Since COVID-19 demonstrates that HCWs in Sindh province’s public hospitals are more likely to become ill due to a lack of safety equipment, an emergency room, beds, and rooms, their work is widely regarded as extremely hazardous. The consequences of this study, which was made possible by the funding received for this study, revealed that the vast majority of HCWs struggled with issues like infection anxiety, burnout, and subpar work. All of these issues originated from subpar resources dedicated to health care and emergency response, as well as from a lack of necessary infrastructure and subpar tools for dealing with health crises. The study found that because of the inadequate emergency facility, HCWs were uncomfortable working in a respiratory infection environment. The HCWs made do with subpar resources when it came to emergency preparedness and equipment. The public hospital in Sindh, Pakistan, can benefit both HCWs and Patients with the right medical essential goods, infrastructure, beds, rooms, and health-hazard treatment facility.

## Limitation

There are several limitations to this study. The first is that the data was gathered from HCWs in Pakistan’s Sindh Province. The second limitation is that the study used a qualitative inductive research design. The study also used semi-structured interviews. As a result, in the future, this study can be conducted in different regions, and the method can be expanded further.

## Suggestion

- The public hospital sector (PHS) in Pakistan’s Sindh province requires updated physical equipment to promote HCW comfortability in terms of patient treatments.
- The public hospital sector authority in the Sindh province of Pakistan must provide the most recent personal safety equipment for healthcare workers’ self-protection.
- The Sindh government must develop public hospital infrastructure, such as a building and rooms.
- HCWs must provide training on the most recent forms of work-health equipment and self-safety in terms of stress work procedures.

## Data Availability

Data for the study is available and will be supplied upon request.
